# Body-selective areas in the visual cortex are less active in children than in adults

**DOI:** 10.3389/fnhum.2014.00941

**Published:** 2014-11-21

**Authors:** Paddy D. Ross, Beatrice de Gelder, Frances Crabbe, Marie-Hélène Grosbras

**Affiliations:** ^1^Institute of Neuroscience and Psychology, University of GlasgowGlasgow, UK; ^2^Brain and Emotion Laboratory, Department of Cognitive Neuroscience, Maastricht UniversityMaastricht, Netherlands

**Keywords:** body perception, fMRI, children, multivariate Bayes, functional development

## Abstract

Our ability to read other people’s non-verbal signals gets refined throughout childhood and adolescence. How this is paralleled by brain development has been investigated mainly with regards to face perception, showing a protracted functional development of the face-selective visual cortical areas. In view of the importance of whole-body expressions in interpersonal communication it is important to understand the development of brain areas sensitive to these social signals. Here we used functional magnetic resonance imaging (fMRI) to compare brain activity in a group of 24 children (age 6–11) and 26 adults while they passively watched short videos of body or object movements. We observed activity in similar regions in both groups; namely the extra-striate body area (EBA), fusiform body area (FBA), posterior superior temporal sulcus (pSTS), amygdala and premotor regions. Adults showed additional activity in the inferior frontal gyrus (IFG). Within the main body-selective regions (EBA, FBA and pSTS), the strength and spatial extent of fMRI signal change was larger in adults than in children. Multivariate Bayesian (MVB) analysis showed that the spatial pattern of neural representation within those regions did not change over age. Our results indicate, for the first time, that body perception, like face perception, is still maturing through the second decade of life.

## Introduction

Perception of signals conveyed by other people’s faces or bodies is still improving in late childhood (Burgund et al., 2002; Johnston et al., 2011; Ross et al., 2012). Along with an individual attainment in social skills and with endocrinological changes, this protracted development may be linked to a shift from one perceptual or cognitive strategy to another (review in Mckone et al., 2012). Functional magnetic resonance imaging (fMRI) studies have revealed that face-selective regions in the occipitotemporal cortex show a particularly long developmental course, taking more than a decade to become adult-like (review in Grill-Spector et al., 2008; Scherf et al., 2011; but see Mckone et al., 2012). In contrast, very few studies have looked at the development of the functional brain response to body perception. As the processing of body cues becomes particularly important during adolescence when social interaction becomes more complex (perception of dominance or aggression for instance), one might expect the cerebral processes involved in basic body perception to also change during this period as is the case for face processing.

Brain areas identified as being specialized for the recognition and interpretation of human form and motion include the extra-striate body area (EBA) located bilaterally in the lateral occipitotemporal cortex, the fusiform body area (FBA), areas in the inferior parietal lobe (IPL) and posterior superior temporal sulcus (pSTS; Downing et al., 2001; Peelen and Downing, 2005; de Gelder, 2006; Grosbras and Paus, 2006; Weiner and Grill-Spector, 2011; Grosbras et al., 2012). Previous fMRI studies have reported activity within those regions when children as young as 7 years old viewed static (EBA and FBA: Peelen et al., 2009; EBA: Pelphrey et al., 2009) or dynamic (pSTS and FBA: Carter and Pelphrey, 2006; Anderson et al., 2013) body stimuli. How this activity differs from that observed in adults is still unclear however, as this has only been tested using samples encompassing a large age-span including pre and post-pubertal individuals (Peelen et al., 2009) or using an index of activation that does not compare directly the height and spatial extent of the activation (Pelphrey et al., 2009). There is also evidence across modalities, of an initial right lateralisation in face (Golarai et al., 2010), voice (Rice et al., 2014) and body (Pelphrey et al., 2009) selective areas, which decreases with age.

Hence, our first goal was to further investigate the recruitment of EBA, pSTS and FBA during body movement perception in pre-pubertal primary-school children as compared to adults in terms of its amplitude and recruited spatial territory. We also wanted to replicate the decreasing right lateralisation effects observed by Pelphrey et al. (2009) (i.e., larger rEBA than lEBA in the children, and vice-versa for the adults). Taking into account the pubertal stage of participants Pubertal Developmental Scale (PDS; Petersen et al., 1988), allowed us to focus on a more homogenous sample than previous studies. Furthermore, using a passive task allowed us to focus on the body-selective areas without any potential interference or modulation from motor or decision making regions (Witt and Stevens, 2013).

Secondly, global level inferences obtained using current univariate approaches may not tell the full developmental story. They negate interactions between voxels, which can only be observed if one looks at patterns of neural representation. For example, (Morcom and Friston, 2012) have shown that while the level of prefrontal activity during a memory task did not change with age, the spatial pattern of neural activity associated with successful encoding was more distributed in older adults, supporting the notion that aging alters the spatial deployment of neuronal activity within specialized functional regions. Similarly, Golarai et al. (2010) showed that a sparse spatial representation best explained their face perception data, and that this spatial pattern did not change over age.

Here we applied a recently developed analytic method that allows multivariate Bayesian (MVB) model comparison across different patterns of activity both within, and across, regions (Friston et al., 2008). Using this approach, we were able to explore how body information is represented in the relevant brain areas. Specifically, using a similar approach to Morcom and Friston (2012) and Golarai et al. (2010), we compared a distributed and a clustered coding hypothesis. Distributed coding would indicate that most voxels within a region of interest (ROI) have little contribution, with a few making a large contribution. Clustered coding on the other hand would indicate that the voxels making the most contribution are clustered together. We then could compare these differences across adults and children to explore whether the spatial pattern of activity within the body-selective regions changes with age. Or, in other words, how the brain neurally represents perceived body movements over age.

In summary we hypothesize, in line with face processing, that the body-selective areas will be right lateralised in children, but this effect will decrease over age. We further hypothesize, contrary to previous work in body recognition, that the neural representation pattern of body movements, along with the height and spatial extent of activation in the body-selective brain areas, will not be ‘adult-like’ in pre-pubescent children.

## Materials and methods

### Participants

Twenty-Seven primary school children were recruited from schools and afterschool clubs in the West End of Glasgow (Scotland). Three children were excluded because of excessive head motion in the scanner. Therefore data from 24 children (aged 6–11 years: *M* = 9.08 years; *SD* = 1.59, 15 females) were included in the analyses. They were all at Tanner stage 1, that is, pre-pubertal, as assessed using the Pubertal Developmental Scale (PDS; Petersen et al., 1988), a sex-specific eight-item self-report measure of physical development (e.g., growth in stature, breast development, pubic hair etc.) filled in by parents. Permission was obtained from the heads of the schools and the managers of the afterschool clubs in order to promote the study. Written consent was also obtained from the children’s parents or guardians before the testing began. All participants understood that participation was voluntary and gave their assent. The study was in line with the Declaration of Helsinki and was approved by the local Ethics Committee. As a comparison group, a sample of 26 adult volunteers (aged 18–27 years: *M* = 21.28 years; *SD* = 2.11, 15 females) from the University of Glasgow also took part.

### Stimuli

We used 45 short video-clips from a set created and validated by (Kret et al., 2011). Each clip depicted one actor, dressed in black against a green background, moving in a socially meaningful manner (e.g., raising fist as if angry, moving shoulders as if disappointed). Six actors were males and 9 females, with each actor recorded 3 times. The videos were recorded using a digital video camera and were edited to two-seconds (50 frames) long clips. The faces in the body videos were masked with Gaussian filters so that only information of the body was perceived (for full details see Kret et al., 2011).

In addition, various clips of non-human moving objects (e.g., windscreen wipers, windmills, metronomes etc.) were taken from the internet. They were cropped to the same size (960 × 540 pixels, 50 frames, 25 fps) as the human videos using Adobe Premiere Pro and a green border was added to make these stimuli as similar as possible to the body stimuli.

Stimuli were organized into blocks of five clips (10 s). To assess the amount of low-level visual motion in each clip, we computed the average change in luminance between consecutive frames. To do so, for each clips we first estimated change in luminance in the background (corresponding to noise level) and for each pairs of frames extracted the number of pixels where the change in intensity was higher than noise. For each clip we computed the average number of pixels with change across the frames. Then we computed the cumulative motion for the five clips in each block. Overall the blocks of non-human clips had slightly more motion than the blocks of body movements clips, although this did not reach statistical significance (*t*_(16)_ = 1.89, CI:(−0.0024:0.0424), *p* = 0.076).

### Design and procedure

#### Data acquisition

We measured brain activity using a 3T fMRI scanner (Tim Trio, Siemens, Erlangen, Germany) equipped with a 32-channels head coil, using standard EPI sequence for functional scans (TR/TE: 2600 ms / 40 ms; slice thickness = 3 mm; in plane resolution = 3 × 3 mm). In addition, we acquired a high-resolution T1-weighted structural scan (1 mm^3^ 3D MPRAGE sequence) for anatomical localization.

Parents/guardians were allowed to sit with their children in the scanning room if they or their child wished (This was the case for 3 subjects). Head motion was restricted thanks to appropriate cushioning. Children were familiarized with the environment and we acquired a 3 min-dummy scan while they watched a cartoon. This allowed us to give them feedback about their head motion and train them to stay still.

#### Main experiment

A MATLAB script using the Psychophysics Toolbox Extensions (Brainard, 1997) was used to present the stimuli. Stimuli were organized into nine blocks of non-human stimuli (10 s; 5 clips), nine blocks of human stimuli and six 10-seconds-long blocks of blank screen as a baseline each presented twice in m-sequence (Buracas and Boynton, 2002). An experimental run lasted 480 s. Stimuli were back-projected onto a screen positioned behind the subject’s head and viewed through a mirror attached to the head-coil. Subjects were instructed to fixate in the center of the screen and were monitored during the scan to make sure they kept their eyes open. They were then probed verbally post scan to ensure they paid due attention to the stimuli. Subjects also participated in another independent 8-min functional scan during the same scanning session, before completing the structural scans.

### Pre-processing

Pre-processing and statistical analysis of MRI data was performed using SPM 8 (Welcome Department of Imaging Neuroscience).^1^ Functional data were corrected for motion by using a two-pass procedure to register the images to the mean of the images after the first realignment. They were then re-sliced with a 4th Degree B-Spline interpolation. Movement correction was allowed up to 2 mm translation or 2 degrees rotation; the three participants who had larger head motion were excluded from the analysis. Functional data were co-registered with the individual 3D T1-weighted scans by identifying AC-PC landmarks manually. These anatomical scans were segmented for different tissue types and transformed into MNI-space using non-linear registration. The parameters from this transformation were subsequently applied to the co-registered functional data. For all analyses, the data were spatially smoothed with a Gaussian kernel (8 mm FWHM). High-pass temporal filtering was applied at a cut off of 128 s to remove slow signal drifts. This motion corrected, normalized and smooth data were then used in the random-effects analyses detailed below.

By normalizing the data from our adults and children into the same stereotactic template, we were able to directly compare the strength and extent of activation across age groups. Several studies examining the feasibility of this approach have found no significant differences in brain foci locations when the brains of children as young as 6 were transformed to an adult template (Burgund et al., 2002; Kang et al., 2003). These findings gave us confidence that there is no confound of brain size in our results.

### Whole brain analyses

A general linear model was created with one predictor for each condition of interest (Body and Non-Body). Head motion parameters were also included as regressors of non-interest. The model was estimated for each participant and individual contrasts (Body vs. Non-Body) were taken to second-level random effect analyses to create group-averages separately for children and adults. For the main group analyses, group was added as a factor in the GLM and the resulting statistical maps are presented at a threshold of *p* < 0.001 uncorrected with a cluster extent threshold of 10 voxels. For group comparisons contrasts are masked by the uncorrected *p* < 0.05 one-way contrasts maps before being reported at *p* < 0.001 uncorrected.

### ROI definition and analysis

We defined six regions of interest (ROIs): bilateral EBA, bilateral FBA and bilateral pSTS. These were derived by taking the set of contiguous voxels within a sphere of radius 8 mm surrounding the voxel in each anatomical region that showed the highest probability of activation in a meta-analysis of 20 studies examining contrasts between moving body and controls in adults (detailed in Grosbras et al., 2012).

To test for differences in activity across ROIs and age group for each participant we extracted the individual peak *t* values as well as the size (in mm^3^) of the activated clusters at three statistical thresholds (*p* < 0.05 FWE corrected (whole brain contrast) *p* < 0.001 uncorrected, *p* < 0.01 uncorrected). These parameters summary statistics were then entered into 2 × 6 mixed design ANOVAs, with Age-Group as between subject factor and ROIs as within subject factor.

### Multivariate bayesian analysis

We investigated the coding activity patterns within the ROIs with an MVB decoding approach (see Friston et al., 2008; Morcom and Friston, 2012). Decoding models that operate on any voxel sets are ill-posed as there are an infinite number of equally likely solutions. MVB gets around this by using constraints (priors) to estimate the voxel weights under different models (Distributed encoding can be described as where individual patterns of activity are expected to contribute sparsely to the decoding; so most voxels make a small contribution, while a few make a large contribution, and a macroscopic region may contain neurons that have different functional properties. Clustered encoding, on the other hand, indicates that the pattern of activity is clustered with smooth local support (defined by a Gaussian with FWHM = 4 mm^3^); so the voxels making the most contribution are clustered together).

Then the set of voxel patterns chosen constitutes a hypothesis about the nature of the mapping between the brain activity (in this case voxel-wise activity in each of our ROIs) and the target variable (our Body > Non-Body contrast from the whole-brain analysis). MVB can therefore decode the neuronal activity pattern of the target variable according to the spatial priors afforded by each model. The evidence for each model can then be treated as a summary statistic and compared to other models using analysis of variance (ANOVA) (Friston et al., 2008). The dependent measure used is the difference in log-evidence of free energy for the chosen model and a null model (one in which there are no patterns and no mapping).

This allowed us to evaluate competing coding hypotheses (distributed vs. clustered as in Morcom and Friston, 2012 or contiguous vs. non-contiguous as in Golarai et al., 2010) within each ROI of both groups. Furthermore, it allowed us to look at the difference between different coding hypotheses across groups.

### Control for potential artifacts

#### Head motion

Three subjects who showed head motion larger than 2 mm in any translation or 2 degrees in any rotation direction were excluded from the analysis. For the remaining participants, rigid body motion parameters were estimated and used to realign each volume to the averaged image. Those motion parameters were included in the general linear model as parameters of non-interest in order to exclude any potential effect on the activation of interest. In addition, independent *t*-tests did not reveal any significant differences in the mean displacement across six axis between adults and children (X: *t*_(48)_ = −1.05, *p* = 0.298; Y: *t*_(48)_ = 1.92, *p* = 0.061; Z: *t*_(48)_ = −1.35, *p* = 0.185; Pitch: *t*_(48)_ = 0.334, *p* = 0.739; Roll: *t*_(48)_ = 0.412, *p* = 0.682; Yaw: *t*_(48)_ = −0.597, *p* = 0.553). We are therefore confident that any potential group differences in fMRI activation are not a by-product of a small group difference in head motion.

#### Variance in BOLD signal and model fit

Further, variance in BOLD signal could also explain any potential difference we observe between children and adults. To account for this confound we compared the standard deviation of the BOLD signal during blank-screen blocks in our six ROIs across age-groups. We found no significant difference between adults and children in any ROI (rEBA: *t*_(48)_ = 1.12, *p* = 0.270; lEBA: *t*_(48)_ = 1.89, *p* = 0.065; rFBA: *t*_(48)_ = 0.70, *p* = 0.489; lFBA: *t*_(48)_ = 1.26, *p* = 0.215; rpSTS: *t*_(48)_ = 0.688, *p* = 0.495; lpSTS: *t*_(48)_ = 1.415, *p* = 0.163).

In addition, we looked at the residual sum of squares of the full model fit in each of the six ROIs across age. This gave us an estimate of noise in our model for each participant. Again, we found no significant difference between adults and children (rEBA: *t*_(28.21)_ = 1.95, *p* = 0.061; lEBA: *t*_(25.49)_ = 1.98, *p* = 0.059; rFBA: *t*_(48)_ = 1.50, *p* = 0.140; lFBA: *t*_(48)_ = −0.749, *p* = 0.457; rpSTS: *t*_(26.52)_ = 0.1.35, *p* = 0.187; lpSTS: *t*_(28.66)_ = 1.618, *p* = 0.117).

These controls give us confidence that any potential differences in fMRI signal change observed between adults and children are due to functional processing of the stimuli, and not simply due to differences in motion, variance in signal, or within subject error in model fit.

## Results

### Whole-brain contrasts

#### Within groups

**Figure 1:**
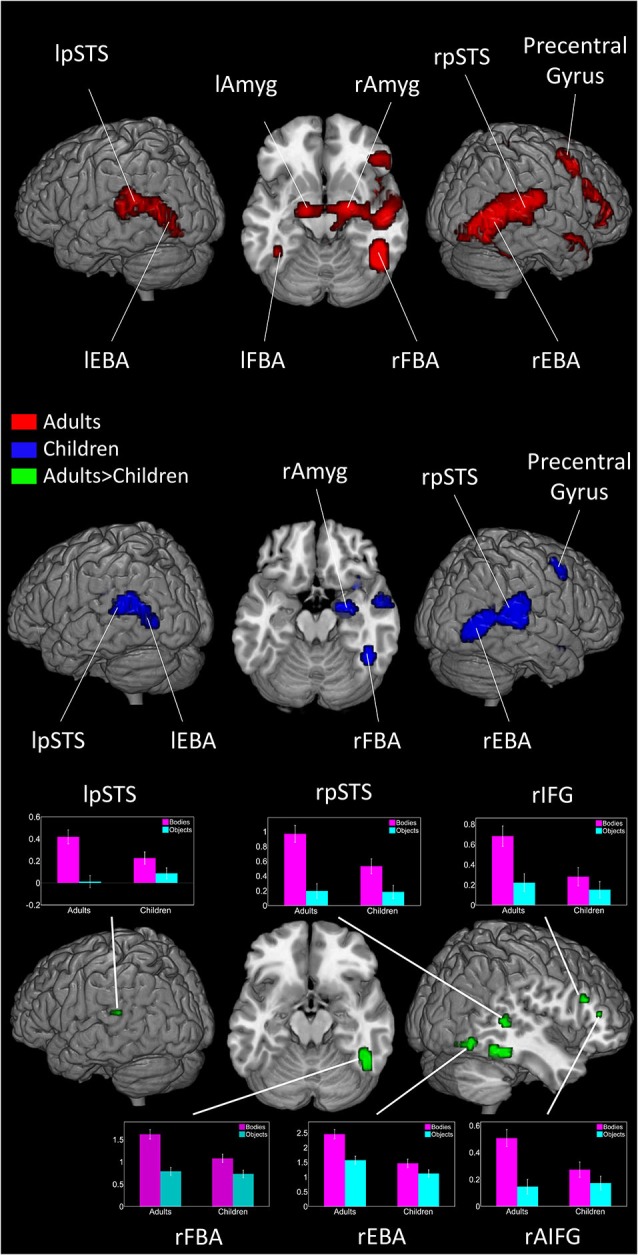
**Brain activity when viewing Bodies > Non-Bodies stimuli in adults and children**. Graph represents effect size of subject’s activity in each area when viewing bodies and objects (threshold at *p* < 0.001 and 10 voxels extent). Error bars represent SEM.

In adults, viewing dynamic bodies compared to viewing dynamic objects activated the bilateral fusiform gyri (including FBA), bilateral occipitotemporal cortices (including EBA), bilateral posterior superior temporal sulci (pSTS), right precentral, right inferior frontal gyrus (IFG), right superior parietal lobule, and bilateral amygdalae. In children, bilateral activity in the occipitotemporal regions and, only in the right hemisphere, fusiform gyrus, pSTS, amygdala and precentral gyrus reached significance level (see Figure 1 and Table 1).

**Table 1:** **Regions activated in a whole-brain group-average random-effects analysis contrasting Bodies > Non-Bodies**.

Region	Adults					Children				
	*x*	*y*	*z*	*t*	mm^3^	*x*	*y*	*z*	*t*	mm^3^
Right Fusiform Gyrus	45	−46	−17	13.00	1890	42	−49	−17	5.51	918
Left Fusiform Gyrus	−42	−40	−20	5.54	567
Right Occipitotemporal	45	−76	−8	11.32	1566	48	−73	4	10.16	1890
Left Occipitotemporal	−45	−76	7	7.59	1728	−54	−67	16	7.00	1512
Right P Superior Temproal Sulcus	57	−43	10	10.10	2187	60	−40	13	7.28	2187
Left P Superior Temporal Sulcus	−63	−49	19	7.42	1458	−51	−55	13	4.00	837
Right Superior Parietal Lobe	30	−49	67	4.55	432
Right Inferior Frontal Gyrus	45	17	28	7.34	2025	36	17	25	4.33	621
Right Precentral Gyrus	48	5	46	8.16	2025	51	2	49	6.18	1323
Right Amygdala	18	−7	−14	7.14	1620	21	−7	−11	6.07	1377
Left Amygdala	−18	−7	−14	6.28	1215
Right Temporal Pole						36	17	−32	4.43	648
Right Precuneus						3	−58	31	5.08	2295
Left Supramarginal Gyrus						−54	−43	31	4.17	972

*(p < 0.001 uncorrected, cluster extent threshold of 10 voxels). Coordinates are in MNI space*.

#### Between groups

Adults showed significantly more activation than children in the bilateral occipitotemporal areas, right pSTS, right fusiform gyrus, bilateral amygdalae, right thalamus and the right IFG.

No region showed higher activity in children than in adults (see Figure 1 and Table 2).

**Table 2:** **Regions activated by whole-brain group-average random-effects analyses contrasting Adults and Children for Bodies > Non-Bodies**.

	*x*	*y*	*z*	*t*	mm^3^
Adults > Children
Right Fusiform Gyrus	45	−52	−17	4.33	1215
Right Occipitotemporal	45	−76	−11	3.91	918
Right P Superior Temporal Sulcus	54	−46	7	3.79	351
Left P Superior Temporal Sulcus	−63	−49	19	3.63	81
Right Anterior Inferior Frontal Gyrus	51	32	10	3.76	81
Right Inferior Frontal Gyrus	54	17	25	3.42	162

*(p < 0.001 uncorrected). Coordinates are in MNI space*.

Children > Adults

*No regions active at given threshold*.

### Region of interest analysis

#### Localization

The average MNI coordinates of the highest positive *t*-value in each of the ROIs were similar in both children and adults, confirming that these regions occupy the same cortical space (see Table 3).

**Table 3:** **Average MNI coordinate of peak *t*-value in all ROIs**.

ROI		x(SD)	y(SD)	z(SD)	*n*
rEBA	Children	49(3)	−72(3)	−1(3)	24
	Adults	50(3)	−72(3)	−1(4)	26
lEBA	Children	−49(3)	−77(3)	−1(3)	21
	Adults	−48(3)	−75(3)	−1(1)	26
rFBA	Children	42(2)	−42(4)	−19(4)	24
	Adults	43(2)	−44(3)	−20(3)	26
lFBA	Children	−40(1)	−45(4)	−16(4)	21
	Adults	−41(2)	−44(3)	−19(3)	26
rpSTS	Children	55(4)	−57(5)	11(3)	24
	Adults	56(4)	−56(5)	11(3)	26
lpSTS	Children	−46(4)	−57(5)	14(3)	22
	Adults	−46(4)	−56(5)	14(3)	26

#### Size

For the three statistical thresholds that we considered (*p* < 0.05 FWE corrected, *p* < 0.001 uncorrected and *p* < 0.01 uncorrected), the proportion of participants showing at least one voxel above threshold was higher in adults than in children in each of the six ROI (see Table 4).

**Table 4:** **No. of subjects showing activity in the six ROIs at each statistical threshold**.

	rEBA	lEBA	rFBA	lFBA	rpSTS	lpSTS
***p* < 0.01**
**Children**
No.	21	14	17	11	17	19
Mean (SD)	1044.9 (712.8)	494.1 (378)	475.2 (540)	291.6 (280.8)	1439.1 (823.5)	747.9 (726.3)
**Adults**
No.	26	22	25	18	25	25
Mean (SD)	1539 (523.8)	939.6 (569.7)	912.6 (548)	380.7 (353.7)	1458 (634.5)	688.5(586)
***p* < 0.001**
**Children**
No.	18	12	12	6	17	13
Mean (SD)	918 (672.3)	342.9 (315.9)	359.1 (502.2)	248.4 (205.2)	1139.4 (820.8)	683 (650.7)
**Adults**
No.	26	21	24	12	24	23
Mean (SD)	1304.1 (612.9)	815.4 (548.1)	696.6 (494.1)	345.6 (251.1)	1247.4 (666.9)	459 (553.5)
***p* < 0.05 FWE corr.**
**Children**
No.	13	4	3	1	8	2
Mean (SD)	432 (558.9)	94.5 (135)	270 (421.2)	81	718.2 (577.8)	54 (37.8)
**Adults**
No.	22	16	14	2	21	8
Mean (SD)	899.1 (594)	548.1 (442.8)	286.2 (210.6)	162 (37.8)	731.7 (637.2)	286.2 (281)

*Mean and standard deviation of activity extent in mm³ is also presented*.

In those participants, the average extent of activity was significantly higher in adults than in children at all three thresholds (Main effect of Age Group: *F*_(1,233)_ = 9.78, *p* = 0.002; *F*_(1,201)_ = 6.0, *p* = 0.015 and *F*_(1,107)_ = 6.12, *p* = 0.015, for threshold *p* < 0.01 uncorrected, *p* < 0.001 uncorrected and *p* < 0.05 corrected respectively). The main effect of ROIs was highly significant at the three thresholds (*F*_(5,233)_ = 18.99, *p* < 0.0001; *F*_(5,201)_ = 12.80, *p* < 0.0001 and *F*_(5,107)_ = 4.36, *p* < 0.0001), and interactions were observed between Age Group and ROIs in all but the most stringent threshold (*F*_(1,233)_ = 14.99, *p* = 0.0001; *F*_(1,201)_ = 7.85, *p* = 0.006 and *F*_(1,107)_ = 0.03, *p* = 0.864). These interactions were driven by children showing, relative to other areas, significantly less extent of activation than the adults in the rFBA (*t*_(48)_ = −3.58, *p* < 0.001) and lEBA (*t*_(31)_ = −2.73, *p* < 0.01) for threshold *p* < 0.01 uncorrected and *p* < 0.001 uncorrected respectively.

#### Peak T value

**Figure 2:**
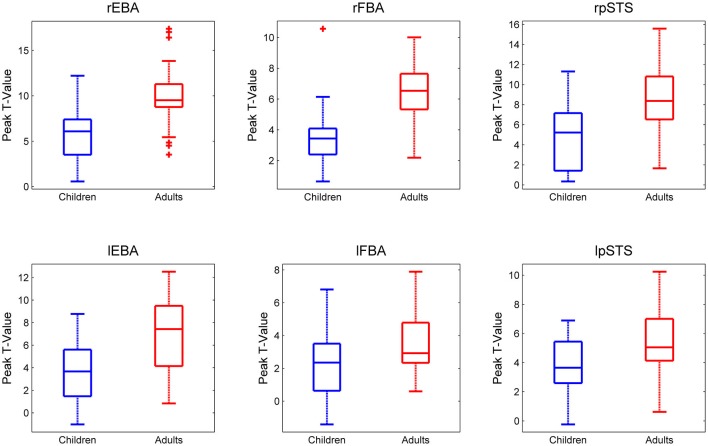
**Peak *t*-values in each ROI for each participant for the Bodies > Non-Bodies contrast**. Children and adults are presented in Blue and Red respectively. It should be noted that the y-axis scales are not homogeneous across ROIs.

The peak *t*-values for adults and children in all six ROIs are presented in Figure 2.

An Age Group × ROI ANOVA revealed a main effect of ROI (*F*_(5,240)_ = 29.05, *p* < 0.0001), a main effect of Age Group (*F*_(1,48)_ = 41.16, *p* < 0.0001) and an interaction between ROI and Age Group (*F*_(5,240)_ = 3.38, *p* = 0.006).

Follow-up (*post hoc*) analyses confirmed that a lack of a significant difference in the lFBA between adults and children was driving this interaction, while all other regions showed significantly higher activity in adults than in children (rEBA: (*t*_(48)_ = −4.77, *p* < 0.001); lEBA: (*t*_(48)_ = −4.22, *p* < 0.001); rFBA: (*t*_(48)_ = −4.91, *p* < 0.001); lFBA: (*t*_(48)_ = −2.41, *p* = 0.095); rpSTS: (*t*_(48)_ = −3.91, *p* < 0.001); lpSTS: (*t*_(48)_ = −2.97, *p* < 0.001).

In addition we verified that this effect was not due to group differences in the processing of moving stimuli: none of the ROIs displayed an age-difference for the contrast Non-Bodies vs. Blank Screen (threshold of *p* < 0.001 uncorrected).

Furthermore, we found no gender differences in any of our ROIs for either age group.

#### Hemispheric differences

Using the peak-t data, a 3 × 2 × 2 mixed design ANOVA with within subject factors ROI (EBA/FBA/pSTS) and Hemisphere (Right/Left), and between subjects factor Age Group (Adults/Children) yielded a main effect of Hemisphere (*F*_(1,48)_ = 83.35, *p* < 0.0001), reflecting greater activity in the right hemisphere than the left. An interaction between Hemisphere and Age-Group (*F*_(1,48)_ = 7.18, *p* = 0.01) was driven by a greater increase in activity in right-hemisphere ROIs over age compared with the left. We found no interaction between ROI and hemisphere (*F*_(2,96)_ = 1.17, *p* = 0.314), and no 3-way interaction (*F*_(2,96)_ = 0.74, *p* = 0.480).

A further 3 × 2 × 2 mixed design ANOVA using a count of the contiguous voxels surrounding each peak yielded similar results. A main effect of Hemisphere (*F*_(1,48)_ = 92.36, *p* < 0.0001) reflected a larger extent of activation in the right hemisphere ROIs. We also observed an interaction between Hemisphere and Age-Group (*F*_(1,48)_ = 7.98, *p* < 0.01) and Hemisphere and ROI (*F*_(2,48)_ = 11.32, *p* < 0.001). *Post-hoc* analyses found this to be caused by a significant increase in activity extent over age in the 3 right lateralised ROIs, but no significant difference in extent activation between children and adults in any of the left lateralised ROIs (rEBA: (*t*_(48)_ = −2.76, *p* < 0.01); rFBA: (*t*_(48)_ = −2.95, *p* < 0.005); rpSTS: (*t*_(48)_ = −2.66, *p* < 0.05); lEBA: (*t*_(48)_ = −1.61, *p* = 0.115); lFBA: (*t*_(48)_ = −1.68, *p* = 0.099); lpSTS: (*t*_(48)_ = 0.239, *p* = 0.812).

### Multivariate bayes analysis

As evidence is only relevant within groups (see above), the main effect of age is meaningless in the following analysis, while the interaction terms allow us to test for differences of models across age groups.

**Figure 3:**
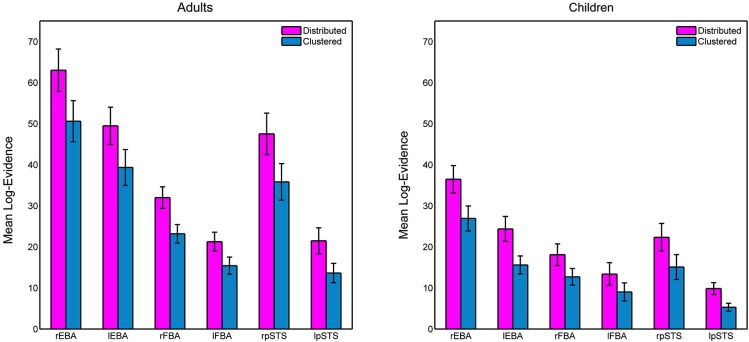
**Mean log-evidence for distributed and clustered models of spatial encoding in the six ROIs in adults and children**. Error bars represent SEM.

Figure 3 shows the group-averaged log-evidence for the competing spatial decoding models in the six ROIs for the children and adults. We performed an ANOVA with within-subject factors spatial prior (Distributed/Clustered) and ROI (rEBA/lEBA/rFBA/lFBA/rpSTS/lpSTS) and between-subject factor Age Group (Children/Adults). We observed a main effect of model (*F*_(1,48)_ = 335.1, *p* < 0.0001), reflecting higher log-evidence for a distributed representation. Although still significant, the difference between distributed and clustered models was smaller in the lFBA relative to other areas, which yielded a significant interaction between model and ROI (*F*_(5,240)_ = 12.2, *p* < 0.0001). We observed no three-way interaction (*F*_(5,240)_ = 0.861, *p* = 0.508), indicating that although the difference in log-evidence between distributed and clustered models differs across ROIs, age produces a purely additive effect to these differences.

## Discussion

We investigated the development of the body-selective areas by comparing brain activity in primary-school children (age 6–11) and adults during passive viewing of body movements compared to object movements. In both groups we observed activity in similar regions to those reported in previous studies using static (Downing et al., 2001; Astafiev et al., 2004; Peelen and Downing, 2005; Taylor et al., 2007) or dynamic (Carter and Pelphrey, 2006; Blake and Shiffrar, 2007; Kret et al., 2011) body stimuli. We observed a right lateralisation of the body-selective regions, and contrary to our hypothesis, extent of activation became more right lateralised in the adults. Finally, children showed, on average, less activation than adults in terms of strength and extent, but we found no difference in the pattern of spatial encoding between children and adults, with both showing more evidence for a distributed model of neural representation.

### Similar “body circuits” recruited in children and adults

A number of studies have confirmed that viewing static or dynamic bodies engages specific regions in the occipito-temporal cortex. Here we observe that these regions are also active in children. With regard to the EBA and the FBA, this confirms previous reports that had used static stimuli (Peelen et al., 2009; Pelphrey et al., 2009). The EBA coordinates are in line with previous reports in adults (Downing et al., 2001; Pelphrey et al., 2009) and children (Peelen et al., 2009; Pelphrey et al., 2009). Our FBA coordinates were also similar to those previously reported by Peelen et al. (2009). Like previous studies we also observed higher activity in the right compared to the left hemisphere in both children and adults. This indicates that the advantage of the right hemisphere in processing socially relevant information is present early on in development. Furthermore, while most previous reports of FBA are limited to the right hemisphere, we also observed activity in the left hemisphere. The age effect, however, was only found to be significant in the right hemisphere ROI.

In addition, we observed activity in the pSTS in both adults and children. As previously stated, the pSTS is implicated in the processing of body related motion (Carter and Pelphrey, 2006; Blake and Shiffrar, 2007). Saygin (2007) showed that when pSTS activity is disrupted, either permanently following a stroke, or temporarily following repetitive trans-cranial magnetic stimulation (rTMS), a person’s ability to perceive bodily motion is significantly impaired. In contrast to EBA and FBA activation (which increases towards bodies compared with objects regardless of whether or not the stimuli are moving), activity in the pSTS is thought to be related only to bodies in motion (Saxe et al., 2004; Pitcher et al., 2011). Furthermore, Candidi et al. (2011) demonstrated that event-related rTMS over pSTS increased the accuracy of a subject in detecting changes of threatening human postures. These results support the notion that along with being involved in the processing of bodily motion, the pSTS is crucial to the detection of socially relevant information concerning others’ actions. Interestingly pSTS activity can be identified with fMRI in children and adolescents during more complex social tasks, such as mentalising, but this activity is still reduced compared to adults (Blakemore et al., 2007).

We also observed activity in both groups in the IFG and inferior parietal lobule (IPL). These regions have been reported during both action observation and action execution (Grèzes and Decety, 2001; Hodzic et al., 2009), which led to suggestions that they contain mirror neurons and represent other people’s actions in relation to one’s own actions (Rizzolatti and Craighero, 2004). It is interesting to observe that this network is already engaged with children when passively viewing body movement (Pfeifer et al., 2007).

Finally, both adults and children showed activity in the amygdala, bilaterally for the adults, while only the right hemisphere cluster reached significance in the children. Amygdala activity is commonly reported in fMRI studies of socially relevant facial expression perception (Kang et al., 2003; Grosbras and Paus, 2006). It has, however, also been implicated in the processing of body movements and posture in the human (Bonda et al., 1996; Hadjikhani and de Gelder, 2003; de Gelder, 2006; Grosbras and Paus, 2006) and non-human (Brothers et al., 1990) brain. While other developmental studies have reported amygdala activity in children during face perception (Killgore and Yurgelun-Todd, 2007; Passarotti et al., 2009), biological motion in point-light displays (Anderson et al., 2013) and inferring mental state from pictures of eyes and stories (Rice et al., 2014), this is the first evidence of children amygdala involvement in processing of full-light dynamic body stimuli.

### Differences in strength and extent of activity in ROIs

Our finding of a significant difference in both peak and extent activity in EBA, FBA and pSTS between children and adults contradicts previous work. Pelphrey et al. (2009) have suggested that the EBA is already as large in children as in adults by the age of 7 years; albeit with a different asymmetry pattern (right dominance in the children but left dominance in adults). This was observed using a very lenient threshold (*p* < 0.01 uncorrected), however, yielding regional activity probably beyond the boundaries of the EBA (with clusters of 38,000 mm^3^). Here we use a different measure in taking the contiguous voxels surrounding the peak in each of our ROIs and this could explain why our results are contrary to Peelen et al. (2009). Instead, using bodies, we replicate the results that Golarai et al. (2010) obtained using faces; we too observed an increase over age in the amount of contiguous voxels in our right hemisphere ROIs, but no significant age difference in the left.

Using a more restricted ROI definition than Pelphrey et al. (2009), Peelen et al. (2009) yielded similar cluster sizes to those we see here. Contrary to the current study, however, they observed no difference between adults and children in the size of FBA and EBA activity (defined by contrast static Body > Tools in the right hemisphere); they even report larger right EBA in children compared to adults when the activity was defined at a threshold *p* < 0.01 or *p* < 0.05 uncorrected. In addition, although they report a trend for increased activity with age, they don’t find any significant difference in peak activity when comparing adults and children. One has to note, however, that the group in their study comprised children and adolescents (age from 7 to 17), with only nine children under the age of 11. Thus differences between children and adults could have been masked by comparatively larger activity in the group of adolescents. Other differences might arise from the use of static photographs as compared to the dynamic stimuli used in our study. A possibility is that the dynamic stimuli engage more of the body-selective areas (Amoruso et al., 2011) and that this difference is stronger in adults than in children. This would then explain the increase over age in strength and extent of activity in the pSTS region and is further supported by reports of a delayed maturation of motion processing compared to static perception in the visual cortex (Bucher et al., 2006; but see Grèzes et al., 2007). Whether the age differences we observe are due to changes in low-level processing or development of higher-level perceptual functions remains to be tested.

Another possibility to be considered is that top-down influences are responsible for the differences observed between children and adults. In other words, although the scans were kept deliberately short in an attempt to minimise a lack of attention in participants, attentional differences could still have arisen from the clips having different significance to adults and children. (Sinke et al., 2010) showed a top-down influence on the body-selective areas when viewing socially meaningful stimuli. These areas, however, were found to be most active when subjects were not attending the stimulus. Therefore to attribute the differences we see here to purely top-down modulation, the adults would have had to be paying less attention to the stimuli than the children.

So, although we cannot rule out the possibility of some top-down influence, we can say with confidence that this is not the sole factor in our observed differences between adults and children.

Taken together with the developmental studies of face perception (Golarai et al., 2007; Scherf et al., 2011), our results suggest that the body-selective areas in the visual cortex do not become adult-like in terms of extent and strength of activity until the second decade of life. How this could be related to the development of social perception abilities like recognizing and interpreting others bodily movements should be explored in future studies.

### A difference in neural representation?

Previous studies showing increase in specificity of cortical activity over age (Carter and Pelphrey, 2006; Grill-Spector et al., 2008) have led authors to suggest different neural representation strategies are used across ages, possibly reflecting different spatial activity patterns within specialized cortical areas (Golarai et al., 2010). Here, using MVB, we directly tested whether, at a functional level, there could be a difference in the way in which the stimuli are being neurally represented. We found strong evidence that a distributed spatial prior gives the best model of neural representation in all ROIs in both adults and children. This means that most voxels in these ROIs are making a small contribution, with a few making a large contribution. The lack of change in representation model over age indicates that the patterns of activity in these regions do not become any more or less functionally distributed between childhood and adulthood. So, even if the amount and extent of neuronal activity when looking at the human body increases throughout childhood into adulthood, the functional representation of the stimuli does not change in terms of spatial encoding. This is in line with work by Golarai et al. (2010) who found, using a similar measure (size of ROI clusters with contiguous or non-contiguous voxels across age), that a sparse spatial representation best explained the data, and that this spatial pattern did not change over age. This is the only aspect of body perception (aside from the extent of activation in the left hemisphere ROIs) that we found to be “adult-like” in children.

## Conclusions

Previous studies had suggested that the body-selective regions in the visual cortex are “adult-like” by the age of 7 years old. Here, using a larger and more homogenous sample, we present evidence for the first time that 11 year-olds still exhibit reduced strength of activation in these areas compared to adults. We also find a significant increase with age in the extent of activation in the body-selective regions, but only in the right hemisphere. Furthermore, using MVB techniques we find evidence that patterns of neural representation do not differ between adults and children. Therefore we conclude that a significant quantitative, but not qualitative maturation occurs during adolescence for processing signals from the human body.

## Conflict of interest statement

The authors declare that the research was conducted in the absence of any commercial or financial relationships that could be construed as a potential conflict of interest.

## Footnotes

^1^See www.fil.ion.ucl.ac.uk/spm
